# The duration of sleep promoting efficacy by dual orexin receptor antagonists is dependent upon receptor occupancy threshold

**DOI:** 10.1186/1471-2202-14-90

**Published:** 2013-08-28

**Authors:** Anthony L Gotter, Christopher J Winrow, Joseph Brunner, Susan L Garson, Steven V Fox, Jacquelyn Binns, Charles M Harrell, Donghui Cui, Ka Lai Yee, Mark Stiteler, Joanne Stevens, Alan Savitz, Pamela L Tannenbaum, Spencer J Tye, Terrence McDonald, Leon Yao, Scott D Kuduk, Jason Uslaner, Paul J Coleman, John J Renger

**Affiliations:** 1 Department of Neuroscience, Merck Research Laboratories, West Point, PA, USA; 2 Department of In Vivo Pharmacology, Merck Research Laboratories, West Point, PA, USA; 3 Department of Pharmacokinetics and Drug Metabolism, Merck Research Laboratories, West Point, PA, USA; 4 Department of Medicinal Chemistry, Merck Research Laboratories, West Point, PA, USA

**Keywords:** Dual orexin receptor antagonist, Suvorexant, Receptor occupancy, Residual effects

## Abstract

**Background:**

Drugs targeting insomnia ideally promote sleep throughout the night, maintain normal sleep architecture, and are devoid of residual effects associated with morning sedation. These features of an ideal compound are not only dependent upon pharmacokinetics, receptor binding kinetics, potency and pharmacodynamic activity, but also upon a compound’s mechanism of action.

**Results:**

Dual orexin receptor antagonists (DORAs) block the arousal-promoting activity of orexin peptides and, as demonstrated in the current work, exhibit an efficacy signal window dependent upon oscillating levels of endogenous orexin neuropeptide. Sleep efficacy of structurally diverse DORAs in rat and dog was achieved at plasma exposures corresponding to orexin 2 receptor (OX_2_R) occupancies in the range of 65 to 80%. In rats, the time course of OX_2_R occupancy was dependent upon receptor binding kinetics and was tightly correlated with the timing of active wake reduction. In rhesus monkeys, direct comparison of DORA-22 with GABA-A modulators at similar sleep-inducing doses revealed that diazepam produced next-day residual sleep and both diazepam and eszopiclone induced next-day cognitive deficits. In stark contrast, DORA-22 did not produce residual effects. Furthermore, DORA-22 evoked only minimal changes in quantitative electroencephalogram (qEEG) activity during the normal resting phase in contrast to GABA-A modulators which induced substantial qEEG changes.

**Conclusion:**

The higher levels of receptor occupancy necessary for DORA efficacy require a plasma concentration profile sufficient to maintain sleep for the duration of the resting period. DORAs, with a half-life exceeding 8 h in humans, are expected to fulfill this requirement as exposures drop to sub-threshold receptor occupancy levels prior to the wake period, potentially avoiding next-day residual effects at therapeutic doses.

## Background

As a primary arousal signal in wake control, orexin signaling is necessary for normal circadian regulation of consolidated wakefulness; exogenous application of orexin neuropeptides or optogenetic activation of orexin neurons induce arousal and genetic loss of orexin signaling is associated with narcoleptic symptoms [1,2]. Endogenous orexin peptide levels oscillate over the course of the day: secretion during waking hours induces a peak in orexin levels late in the active phase, and rapidly decreasing activity precedes a nadir in levels during the inactive phase [3,4]. Orexin-A (OX-A) and -B (OX-B) are processed peptide products of the *HCRT* (*hypocretin*) gene, which encodes the prepro-orexin peptide [5]. OX-A has similar affinity for both orexin-1 and -2 receptors (OX_1_R and OX_2_R, respectively), while OX-B selectively favors OX_2_R binding [6]. Exogenous administration of OX-A is most effective in promoting wakefulness when applied during inactive-phase periods when the endogenous levels are normally low [7-9]. Conversely, blocking orexin activity with dual orexin receptor antagonists (DORAs) is most easily measured during the active phase [10-12].

Therapy targeting insomnia requires defined pharmacodynamic timing. Unlike most other central nervous system therapeutics, compounds utilized in the therapeutic treatment of insomnia will, ideally, promote sleep throughout the resting period, maintain normal sleep architecture, and avoid residual effects upon waking, thus allowing for full wakefulness during the subsequent active period. This time-limited efficacy requires pharmacokinetic exposure and sufficient central receptor occupancy levels to drive sleep efficacy when desired, and reduced compound exposure to avoid residual effects upon waking. This limitation of duration of activity makes the half-life (T_1/2_) of γ-aminobutyric acid (GABA-A) receptor modulators a key defining feature of their clinical utility. Next-day/carry-over effects, in particular, are well-documented undesirable mechanism-related adverse effects of conventional therapies with long half-lives [13,14]. The current standards of care, benzodiazepine and non-benzodiazepine GABA-A receptor modulators, can also be associated with bothersome morning sedation and cognitive residual effects. In contrast, the current data on DORAs suggest that these compounds induce physiological sleep and demonstrate only mild residual effects despite relatively long plasma T_1/2_[10,12,15-18]. Preclinical studies of compounds with balanced potency at OX_1_R and OX_2_R, such as DORA-22, an analog of MK-6096 [2,18,19], demonstrate little or no effect on electroencephalogram (EEG) spectral frequency distribution, suggesting that blockade of orexin-mediated wakefulness allows sleep to occur that is similar to that measured in vehicle-treated controls [20].

Pharmacologically, the duration of sleep-promoting efficacy depends not only upon a compound’s mechanism of action, but also upon its pharmacokinetics, receptor-binding kinetics and potency, as these properties dictate the timing of receptor occupancy required for *in vivo* effects important for a compound’s overall activity and clinical utility. Clearly, rapid central nervous system penetration is essential for sleep-onset efficacy, and a moderate pharmacokinetic T_1/2_ and sufficiently high receptor occupancies are required for sleep maintenance throughout the desired resting period. However, to avoid next-day residual effects, compound levels must drop below exposures required for minimum effective receptor occupancy by the onset of wakefulness to avoid residual sleep effects. In principle, compounds that are effective at a low receptor occupancies are challenged to restrict sleep promoting effects to the resting phase, compared to mechanisms that require a higher occupancy threshold that are less likely to exhibit carry-over effects for a given pharmacokinetic profile and T_1/2_. Here, we characterize the pharmacokinetic and receptor occupancy properties underlying the timing of efficacy elicited by structurally distinct DORAs including DORA-12 and DORA-22, close analogs of suvorexant and MK-6096 whose chemical properties have been described previously [19,21,22]. The ensemble of pharmacokinetic, receptor-binding kinetics and pharmacodynamic properties of DORAs appear advantageous to restricting the wake-inhibition efficacy of these compounds to the resting phase.

## Results

### OX-A levels are associated with wakefulness across species

**Figure 1:**
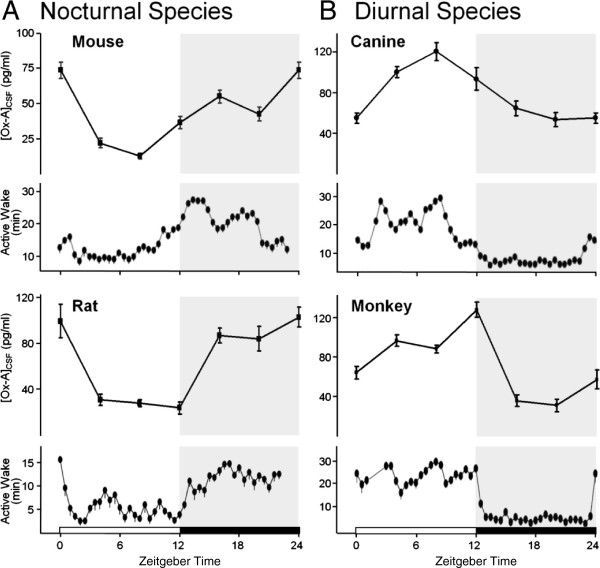
**Active wake closely follows oscillating OX-A levels across preclinical species.** The time course of OX-A levels in CSF and active wake were highest during the dark phase in nocturnal rodents **(A)** and during the light phase in diurnal species **(B)**. Mean OX-A levels and time spent in active wake are plotted ± standard error of the mean (SEM) over a 24-h period (dark period shaded) for 6-h and 30-min intervals, respectively. OX-A levels were determined by meso-scale immunoassay at 6-h time points in mice (n = 3; 50 pooled CSF samples each), rats (n = 8 samples each), dogs (n = 8 samples each), and rhesus monkeys (n = 8 samples for ported animals, each). Mean time in active wake under baseline conditions was determined by EEG from telemeterized mice (6 days, n = 7), rats (6 days, n = 7), dogs (10 days, n = 6), and rhesus monkeys (5 days, n = 7).

To more precisely define the timing of changes in orexin levels, their relationship with arousal and therefore the available signal window for the DORAs over the course of the circadian day, OX-A levels in cerebrospinal fluid (CSF) were determined by meso-scale immunoassay and correlated with baseline active wake as measured by polysomnography (PSG) in nocturnal and diurnal preclinical species. In nocturnal mice and rats, OX-A levels progressively drop during the inactive period, reaching a nadir in the second half of this period, and accumulate over the course of the dark (active) period (Figure 1A). Mean time in active wake generally mimics these OX-A levels. In diurnal dogs and rhesus monkeys, OX-A levels build during the light (active) period and fall during the resting period, coincident with increased arousal during the day and decreased wakefulness at night (Figure 1B). These observations predict that DORAs will have maximum efficacy in the late dark phase of nocturnal species and the late light phase of diurnal animals which correlates to the active periods of both types of species.

### DORA efficacy in rats is most salient during the active phase

**Figure 2:**
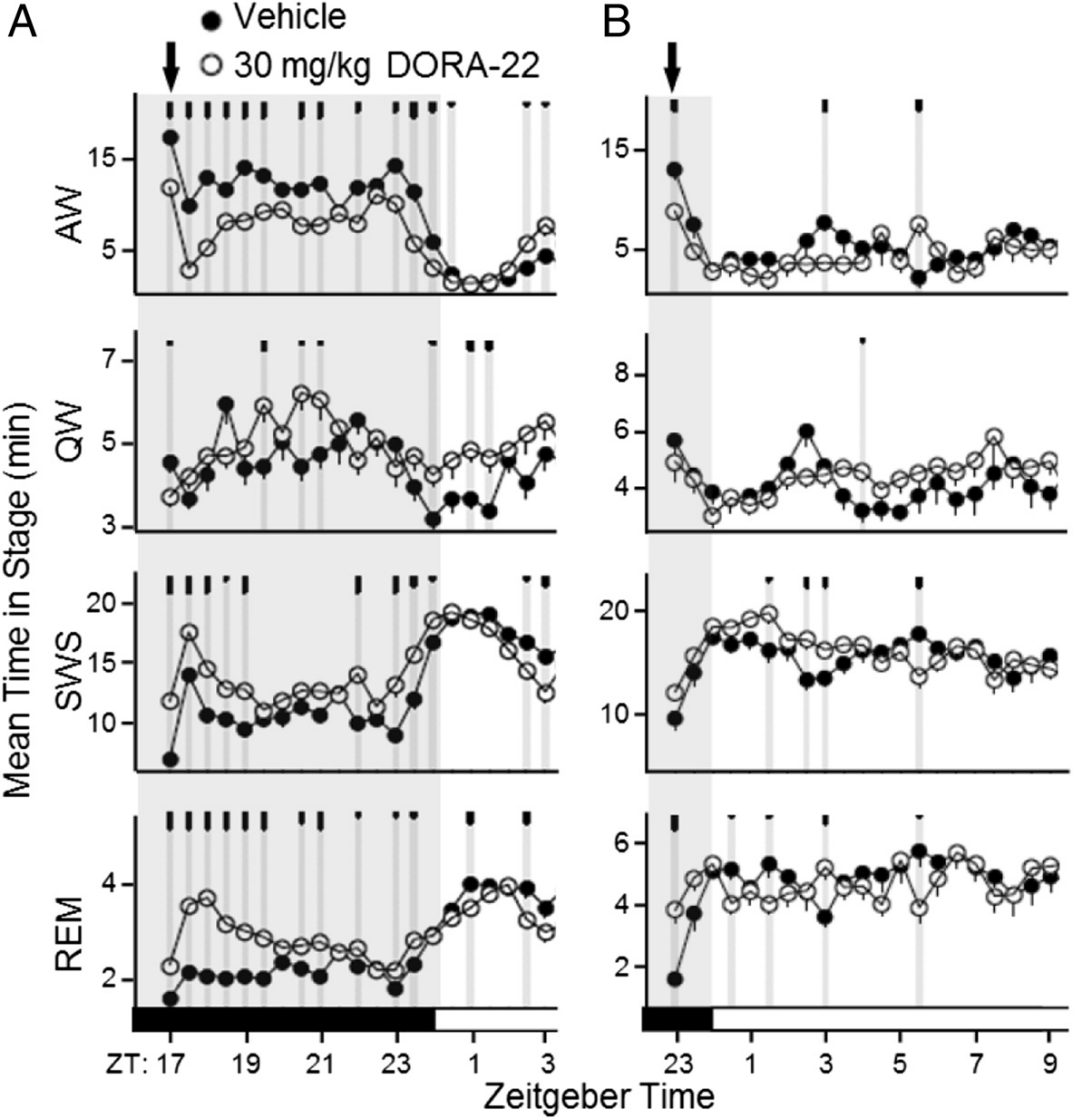
**Sleep-promoting effects of DORA-22 in normal rats are diminished during the inactive phase.** Vehicle (20% Vitamin E TPGS, p.o.) and DORA-22 (30 mg/kg) were administered to rats during the mid-active phase (arrow, ZT 17:00; n = 13) **(A)** or 1 h before the inactive phase (arrow, ZT 23:00; n = 8) **(B)** in a balanced cross-over design. Values represent mean time in sleep stage over 30-min intervals over 3 days of consecutive treatment. Gray shading denotes the dark or active period. AW, active wake; QW, quiet wake. Time points at which significant differences exist between vehicle and DORA-22 responses are indicated by gray vertical lines and tick marks (short, medium, long marks: P < 0.05, 0.01, 0.001, respectively).

The sleep-promoting effects of DORAs were greatest during the active phase when orexin levels were highest in normal rats entrained to a 12:12 light:dark cycle. When administered during the mid-active phase, DORA-22 (30 mg/kg), an MK-6096 analog structurally distinct from suvorexant and DORA-12, promoted sleep characteristic of a DORA: active wake was attenuated for up to 8 h, and both delta and REM sleep were increased for up to 6 h following treatment (Figure 2A). The magnitude of wake-reducing activity was diminished when normal rats were administered DORA-12 just prior to the inactive phase onset and was accompanied by small variable increases in delta and rapid eye movement (REM) sleep (Figure 2B). Importantly, changes induced by DORA-22 administered just prior to the inactive phase trend in the same direction as that seen in vehicle-treated animals progressing from the active to the inactive phase; namely, diminished active wake, increased slow wave sleep (SWS), and small fluctuations in REM sleep.

### DORA-induced efficacy occurs at OX_2_R occupancies exceeding 65%

**Figure 3:**
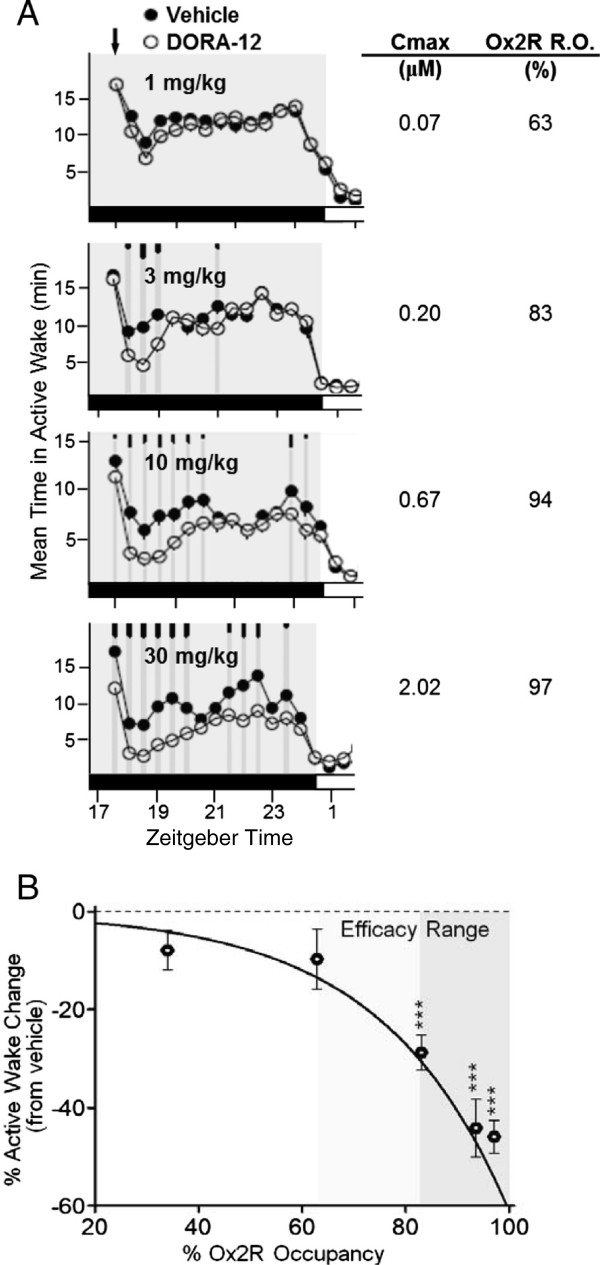
**Sleep efficacy of DORA-12 in rats is associated with OX**_**2**_**R occupancies between 63 and 83%. A**. Attenuation of mean time in active wake in rats in response to DORA-12 (1 mg/kg [n = 14], 3 mg/kg [n = 14], 10 mg/kg [n = 8], and 30 mg/kg [n = 14) relative to vehicle (20% Vitamin E TPGS, p.o.) dosed mid-active phase (arrow). Gray shading, dark period. Time points at which significant differences exist between vehicle and DORA-12 responses are indicated by gray vertical lines and tick marks (short, medium, long marks: P < 0.05, 0.01, 0.001, respectively). Maximum plasma values (C_max_) and corresponding occupancy values determined in satellite animals are listed to the right. **B**. The mean percent change (± SEM) in active wake quantified from individual PSG recordings from ‘A’ versus percent OX_2_R occupancy determined in rats (satellite animals). Light and dark shading indicates potential and definitive occupancy ranges. *** P < 0.001 (t-test) difference in percent active wake change versus baseline.

A dose response for DORA-12, a structural analog of suvorexant, was evaluated in rat PSG studies to determine the relationship between sleep-promoting efficacy as measured by active wake reduction and OX_2_R occupancy. Dose-dependent attenuation of active wake was observed with DORA-12 (Figure 3A), which also resulted in corresponding increases in non-REM and REM sleep. Sleep-promoting efficacy induced by each dose of DORA-12 was quantified as a percent change in active wake relative to the vehicle-induced response over 2 h following treatment.

Separately, the relationship between OX_2_R occupancy relative to plasma exposure was determined by administering DORA-12 intravenously (i.v.) over a range of doses to transgenic rats expressing human OX_2_R to determine a full occupancy curve over a wide range of plasma concentrations in order to define the maximal occupancy against which raw occupancy values could be normalized. This relationship was then used to determine the OX_2_R occupancy in PSG experiments from measured plasma concentrations. The maximum plasma concentration (C_max_) observed in satellite animals dosed in parallel with those used in PSG experiments were used for these analyses since C_max_ occurs during the first two hours after treatment. Increased OX_2_R occupancy, achieved at higher doses, was accompanied by progressively greater attenuation of active wake (Figure 3B). This same pattern was also observed for suvorexant and structurally distinct compounds such as MK-6096 and DORA-22. Generally, compound-induced attenuation of active wake became salient and significant at OX_2_R occupancies between 63 and 83%. While 0.3 and 1 mg/kg doses of DORA-12 marginally reduced active wake, this change became highly significant at 3 mg/kg, where plasma levels reached 0.20 μM, corresponding to 83% OX_2_R occupancy (Figure 3, Table 1). For comparison, efficacy as determined by active wake reduction, plasma exposure, and OX_2_R occupancy in rats are presented for each dose of suvorexant and DORA-12 in Table 1.

**Table 1:** **Efficacy as measured by active wake reduction by suvorexant and DORA-12 relative to plasma exposure and OX**_**2**_**R exposure in rats**

**Compound**	**Dose (mg/kg)**	**Plasma (nM)**	**OX**_**2**_**R Occ**^**a **^**(% max**^**b**^**)**	**Active wake reduction**^**c **^**%**
Suvorexant	10	1600.0	92.6	-29.0***
	30	2100.0	94.3	-79.6**
	100	5100.0	97.6	-79.9**
DORA-12	0.3	20.2	34.0	-7.9
	1	67.4	62.8	-9.7
	3	202.2	83.0	-28.8***
	10	674.0	93.9	-44.2***
	30	2022.0	97.1	-45.9***

^a^Occ, occupancy.

^b^Normalized occupancy was determined over a range of doses and fit to a one-sided binding hyperbolic curve to determine the maximum occupancy. OX_2_R occupancies corresponding to plasma values at indicated doses were calculated based on the normalized curve for each compound.

^c^Mean percent change in active wake relative to vehicle in the 2 h following treatment (**, ***, p < 0.01, 0.001, population t-test).

**Figure 4:**
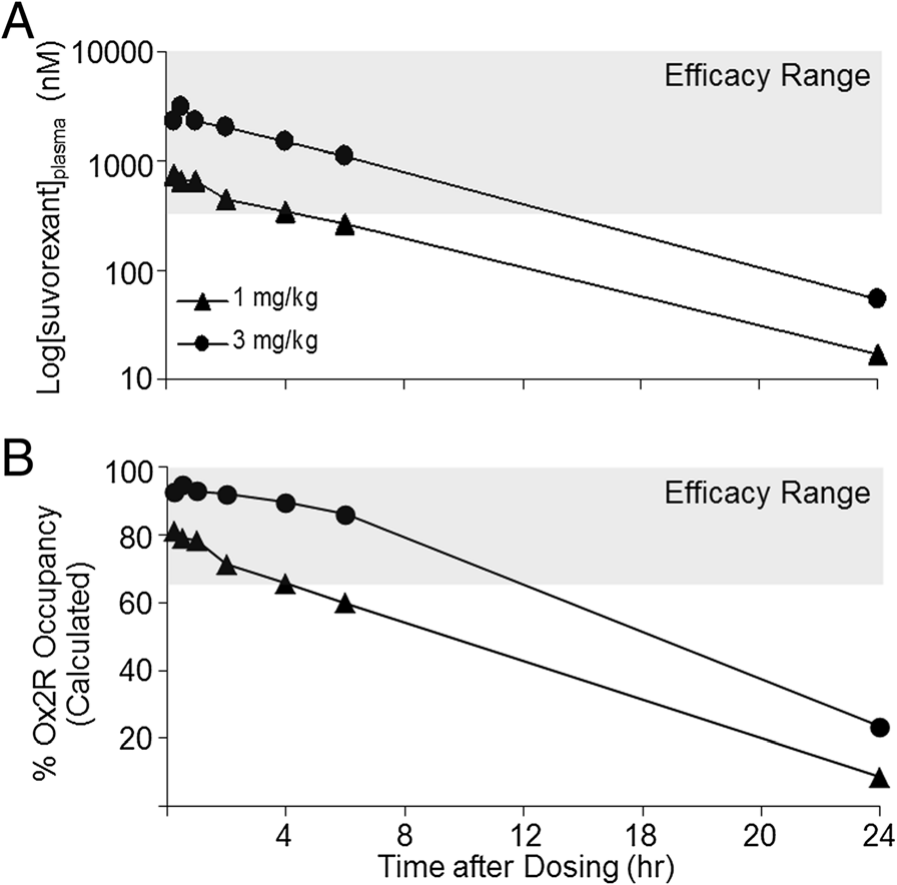
**Suvorexant efficacy in dogs corresponds to plasma exposures > 342 nM and OX**_**2**_**R occupancies > 65%. A**. Suvorexant levels in satellite animals treated with suvorexant (1 or 3 mg/kg; p.o. in 20% Vitamin E TPGS) (n = 3, each time point). **B**. Calculated OX_2_R occupancy based on rat occupancy values, and normalized for free fraction of compound in rat (1.4%) and dog (1%). Gray shading indicates time points at which active wake was significantly attenuated in dogs treated with suvorexant (1 or 3 mg/kg), the plasma and occupancy range at which efficacy has been observed [12].

It was previously shown that suvorexant attenuated active wake in dogs in a dose-dependent manner with corresponding increases in non-REM and REM sleep: the effects of 1 mg/kg on active wake were observed for up to 4 h, and 3 mg/kg responses for up to 7.5 h [12]. Re-examination of the time course of plasma exposures indicated that suvorexant efficacy was observed at time points when plasma concentrations were in excess of 0.342 μM (Figure 4A), a plasma exposure corresponding to 65.7% OX_2_R occupancy calculated from rat occupancy values normalized for both rat and dog unbound plasma values (1.4 and 1.0%, respectively). Time points showing active wake reduction following administration of suvorexant (1 and 3 mg/kg) coincided with plasma exposures that were calculated to result in ≥65% OX_2_R occupancy (Figure 4B), consistent with the range of effective occupancy observed in rats.

### OX_2_R binding kinetics contribute to the timing of DORA receptor occupancy

**Figure 5:**
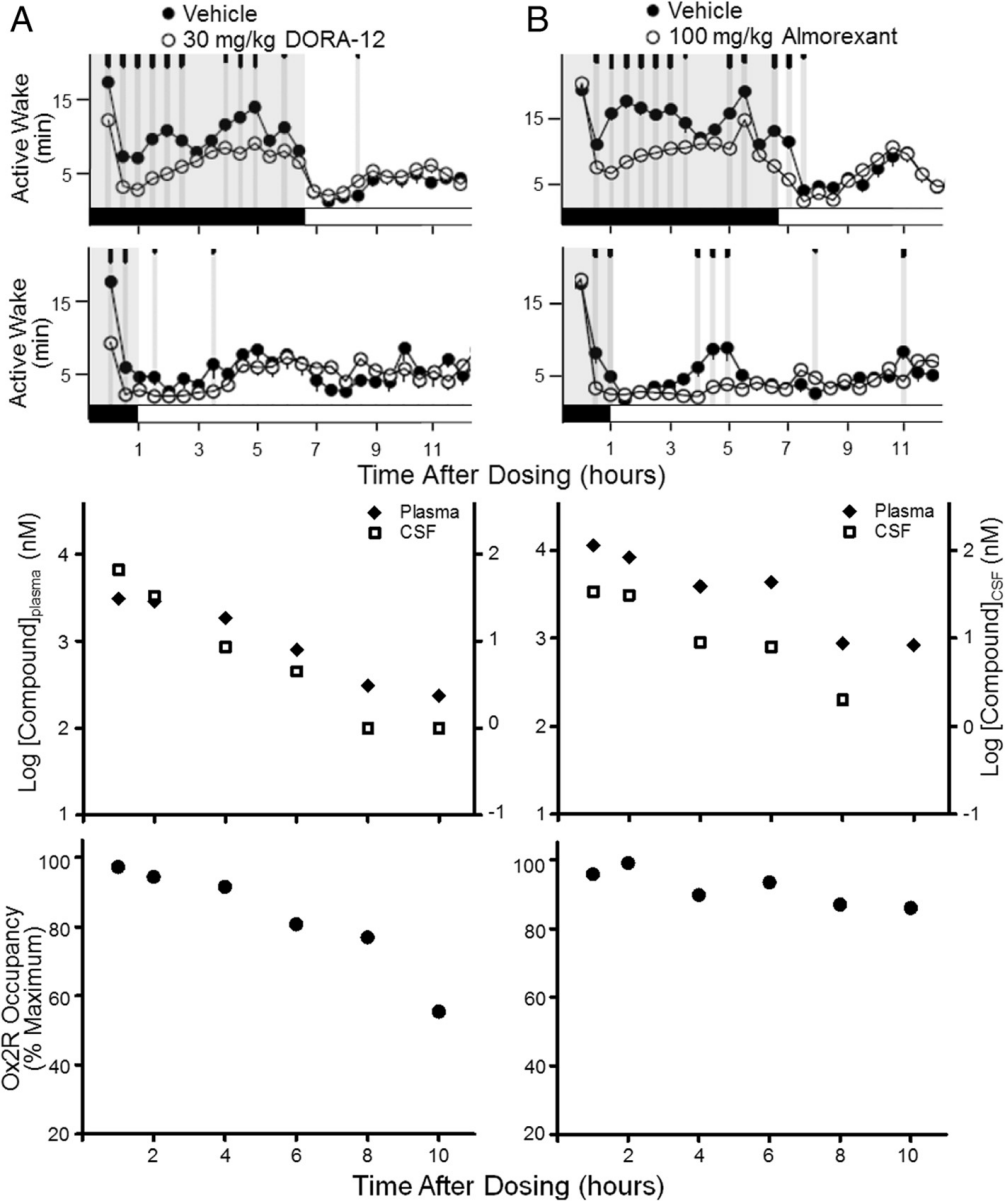
**Time course of DORA-12 and almorexant OX**_**2**_**R occupancy and efficacy in rats. A** (upper panels). The time course of DORA-12 (30 mg/kg) effects on active wake was determined in rats by telemetry PSG relative to vehicle (20% Vitamin E TPGS, p.o.) following treatment in active (n = 14) or inactive phase (n = 7). Time points at which significant differences exist between vehicle and DORA-12 responses are indicated by gray vertical lines and tick marks (short, medium, long marks: P < 0.05, 0.01, 0.001, respectively). **B** (upper panels). Effects of almorexant (100 mg/kg) on active wake relative to vehicle following treatment in active (n = 14) or inactive phase (n = 14). Lower panels. Plasma and CSF levels as well as OX_2_R receptor occupancy of DORA-12 **(A)** and almorexant **(B)** following inactive phase treatment (one male, one female animal per time point). Plasma, CSF, and occupancy levels were determined in the same animals, in satellite to PSG experiments.

To determine the influence of OX_2_R binding kinetics on receptor occupancy and sleep-promoting efficacy, the time course of *in vivo* effects in response to DORA-12 was compared with that of almorexant, a compound known to exhibit exceedingly slow dissociation from OX_2_R. Almorexant is well documented to have slow binding kinetics relative to other DORAs [18,23]. Depending upon the method and competing ligand used, the T_1/2_ of almorexant for association with OX_2_R ranges from 28 to 162 min and from 70 to 268 min for dissociation (the later values obtained in current experiments comparing 6 different DORAs in Table 2). DORA-12 (30 mg/kg) and almorexant (100 mg/kg) were administered both during the mid-active phase and just prior to the inactive phase, a therapeutically relevant dosing time, in rats. DORA-12 significantly attenuated active wake for approximately 6 h following dosing during the active phase and up to 3.5 h after treatment at the inactive phase onset (P <0.05 through 0.001; see Figure 5A, upper panels). Almorexant-dependent reductions in active wake were seen for up to 7.5 h after treatment during the active phase and for up to 11 h following inactive-phase treatment (Figure 5B, upper panels).

**Table 2:** Orexin 2 receptor binding kinetic parameters for DORAs

**Compound**	**K**_**d **_**(nM)**	**T**_**1/2 **_**ON (min)**	**K**_**ON **_**(mol**^**-1**^ **· min**^**-1**^**)**	**T**_**1/2 **_**OFF (min)**	**K**_**OFF **_**(min**^**-1**^**)**
Almorexant	0.048	162^a^	1.58 × 10^7^	268	2.696 × 10^-3^
Suvorexant	0.401	80.1	7.63 × 10^6^	89.4	7.784 × 10^-3^
DORA-12	0.157	57.5	2.77 × 10^7^	232	3.154 × 10^-3^
MK-6096	0.338	62.9	1.69 × 10^7^	118	6.754 × 10^-3^
DORA-22	0.834	24.7	9.19 × 10^6^	37.8	1.878 × 10^-2^
SB-649868	0.163	37.0	2.56 × 10^7^	57.0	1.258 × 10^-2^

^a^Almorexant T_1/2_ ON represents an underestimate as almorexant was applied at concentrations 3-fold above its K_d_ in order to measure its kinetics alongside other DORAs.

K_d_, binding constant; K_OFF_, dissociation constant; K_ON_, association constant; T_1/2_ OFF, half-life off-rates; T_1/2_ ON, binding half-life.

Plasma and CSF levels of DORA-12 and almorexant, as well as OX_2_R occupancy were determined in satellite rats following treatment at the onset of the inactive phase. As seen in Figure 5A (lower panels), DORA-12 plasma and CSF levels began to fall immediately after dosing, with corresponding decreases in OX_2_R occupancy to 77% of maximum at 8 h, coincident with the cessation of sleep-promoting effects. OX_2_R occupancy by almorexant, however, remained elevated at 10 h, even though levels within the CSF were undetectable at this time point (Figure 5B, lower panels). The persistence of OX_2_R occupancy by almorexant despite disappearing levels of the compound in the plasma and CSF is consistent with its extremely slow receptor-binding kinetics relative to other DORAs including suvorexant, MK-6096, DORA-22 and SB-649868 (Table 2). Together these results indicate that binding kinetics have the capacity to influence receptor occupancy and sleep promoting efficacy, particularly for compounds with excessively slow kinetic parameters.

### Next-day sleep, qEEG, and quantitative effects in rhesus monkeys

To evaluate the potential for both GABA-A receptor modulators and DORA-22 to promote sleep through the resting phase and into the subsequent active phase, rhesus monkeys were administered these compounds 2 h before lights out. The doses, diazepam at 10 mg/kg, eszopiclone at 10 mg/kg, and DORA-22 at 30 mg/kg, were selected based on their similar potential to promote sleep following active-phase dosing. Quantified over 2 h, these treatments have previously been found to attenuate active wake by 38.4, 55.0, and 40.1 min relative to vehicle, respectively [24].

**Figure 6:**
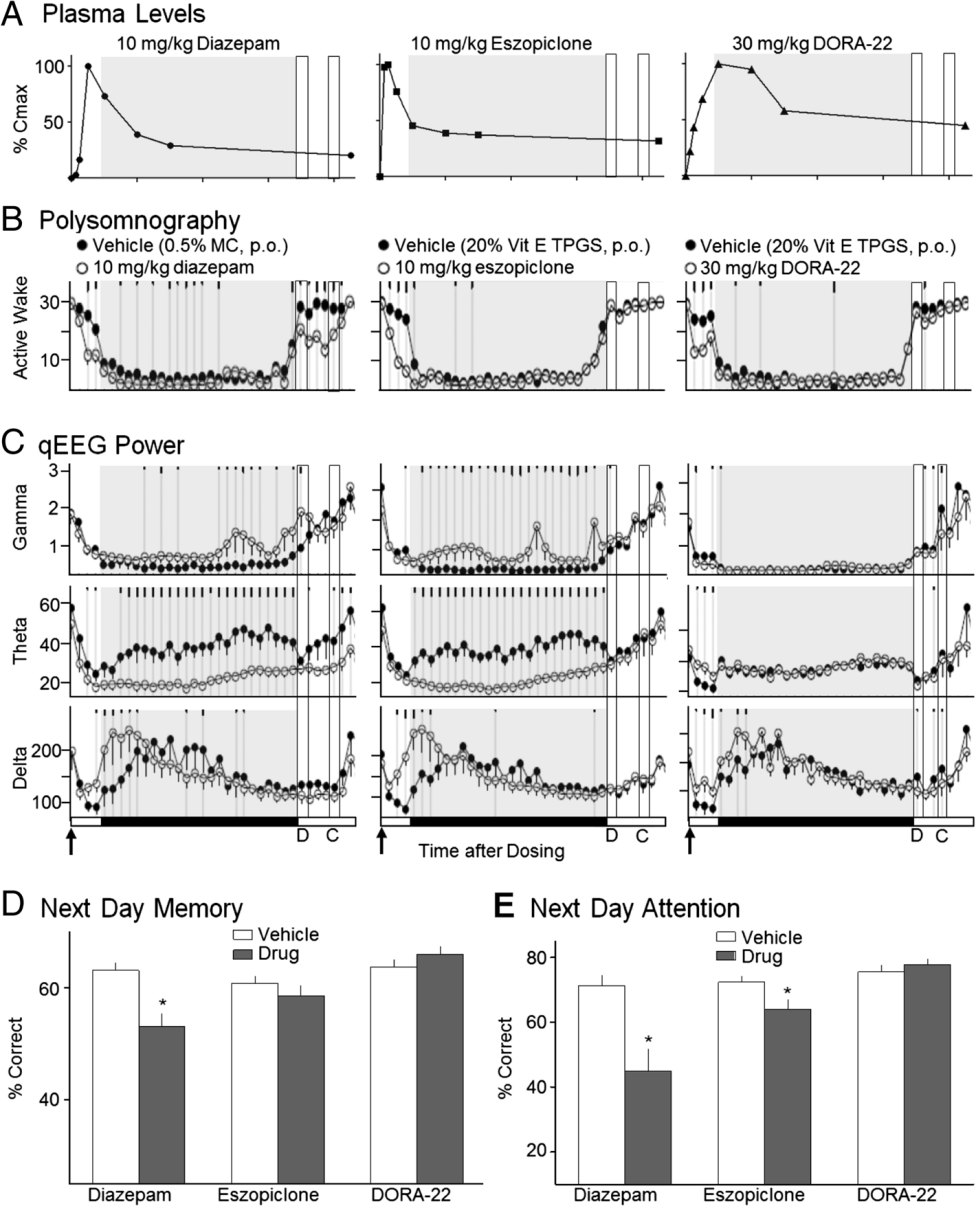
**Diazepam and eszopiclone, but not DORA-22, exhibit next-day effects impacting cognitive performance in monkeys.** Monkeys were treated (arrow) with sleep-promoting doses 2 h before the 12-h dark/inactive phase (gray shading): diazepam, 10 mg/kg, p.o. in 0.5% methylcellulose; eszopiclone, 10 mg/kg, in 20% Vitamin E TPGS; DORA-22, 30 mg/kg, in 20% Vitamin E TPGS. Memory and attention were evaluated by DMS **(D)** and SCRT **(C)** tasks 14 and 16 h after dosing, respectively. **A**. Time course of plasma levels after dosing (0, 0.25, 0.5, 1, 2, 4, 6, 17 h; n = 2, diazepam and eszopiclone; n = 3, DORA-22 [17 h, n = 8]). Normalized plasma levels relative to maximum (C_max_: diazepam 775 nM [1 h]; eszopiclone 1680 nM [0.5 h]; DORA-22 134 nM [2 h]) compare compound levels on the same y-axis scale. **B**. Mean time in active wake (+ SEM) determined by polysomnography in 30-min intervals after treatment (vehicle, closed symbols; compound, open symbols). Significant differences versus vehicle are indicated by gray vertical lines and tick marks (short, medium, long: P < 0.05, 0.01, 0.001, respectively). **C**. Mean qEEG power in gamma (35–100 Hz), theta (4–8 Hz), and delta (0.5–4 Hz) frequency bands following treatment. **D**. DMS measure of memory at 14 h after treatment. Data are mean proportions of completed trials during which a correct choice was made (+ SEM; n *=* 16, 16, and 15, respectively). Random responding, 25%. Significant differences from vehicle: * P < 0.05 (repeated measures ANOVA). **E**. SCRT evaluation of attention at 16 h after treatment. Data are the mean proportions of completed trials during which a correct choice was made following short duration cues (+ SEM; n *=* 16, 16, and 5, respectively). Random responding, 10%. Significant differences from vehicle: * P < 0.05.

Diazepam, eszopiclone, and DORA-22 produced characteristic plasma-exposure profiles (Figure 6A). The anxiolytic diazepam exhibited a peak at 1.0 h (775 nM), eszopiclone induced a more rapid C_max_ at 0.5 h (1680 nM), and DORA-22 a more delayed exposure profile (134 nM at 2 h). All three compounds significantly attenuated active wake in the 2 h after dosing before the onset of the inactive phase (Figure 6B). Unlike eszopiclone and DORA-22, diazepam significantly attenu-ated active wake at time points 7.5 h into the inactive phase and 3 h into the subsequent active phase. Despite the presence of measurable eszopiclone and DORA-22 plasma levels 17 h after treatment, no consistent attenuation of active wake was observed in response to either compound the morning after treatment.

Quantitative EEG analysis performed coincident with PSG revealed time-dependent and qualitative differences between the effects of these compounds that were not evident in measurements of active wake. All three compounds increased delta frequency qEEG power in advance of the normal increase in delta power observed during the resting phase after administration of vehicle (Figure 6C, bottom panels). However, both diazepam and eszopiclone increased high-frequency gamma power and substantially decreased theta power throughout the inactive period, while DORA-22 had essentially no effect on these measures (Figure 6C, top and middle panels). The effects of diazepam persisted into the subsequent active period while eszopiclone-induced qEEG changes ceased abruptly upon lights on (at which point cognitive tests were performed). The relevance of compound-induced qEEG changes occurring during cognitive testing intervals is currently unknown.

In order to better understand the consequences of compound-induced sleep and qEEG effects that persist though the inactive period, cognitive memory and attention parameters were evaluated in the same rhesus monkeys, the morning after treatment. Memory was evaluated in the delayed match to sample (DMS) task performed at lights on (14 h after dosing), while attention was evaluated in the serial choice retention time (SCRT) test administered 2 h later (16 h after dosing; see Figure 6C, bottom panels, D and C time points). Significant deficits in recognition memory, as assessed by DMS, were observed the morning after treatment with diazepam (10 mg/kg) but not following eszopiclone (10 mg/kg) or DORA-22 (30 mg/kg) relative to their respective vehicle treatments (Figure 6D). These results are consistent with diazepam-dependent active wake attenuation persisting into the active period. Despite the presence of measurable levels of this DORA-22 three hours after the DMS task was performed (Figure 6A; 61 nM), no attenuation of in performance was observed. In SCRT, both diazepam and eszopiclone elicited significant deficits in the percentage of correct responses to cues of the shortest duration relative to vehicle-treated controls (P < 0.05 for both) (Figure 6E). In contrast, deficits in next-day attention were not observed with DORA-22.

### Suvorexant exposure time course in humans

**Figure 7:**
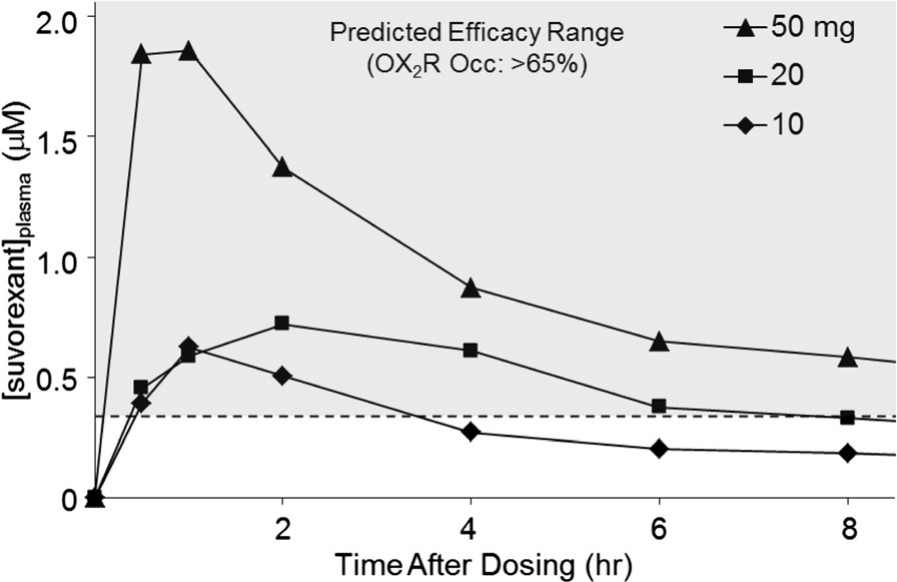
**OX**_**2**_**R occupancy by suvorexant sufficient for sleep-promoting efficacy: restricted to 8 h following treatment.** The time courses of plasma levels of suvorexant in humans collected in Phase 1 clinical trials following administration of suvorexant (indicated doses) are shown relative to the plasma values (0.33 μM) calculated to correspond to the exposure required for 65% OX_2_R occupancy (dashed line). The gray shaded area represents the predicted efficacy range based upon this value. occ, occupancy.

Plasma samples obtained in an Phase 1 clinical evaluation of suvorexant exhibited a common exposure profile, with a time to maximum plasma concentration (T_max_) of 3 h or less (Figure 7). Human free plasma levels of suvorexant sufficient for 65% OX_2_R occupancy were calculated based on extensive transgenic rat occupancy data and normalized for free fraction (rat: 1.4%; human: 1.0%). The human plasma concentration of suvorexant calculated to correspond to 65% OX_2_R occupancy is 0.33 μM (free + bound). Observed clinical suvorexant mean plasma concentrations fell below the levels predicted to correspond to 65% OX_2_R occupancy, where sleep-promoting efficacy is predicted, by the 8 h post-dose time point for both the 10 and 20 mg therapeutic doses. Together this analysis indicates that the sleep promoting effects of suvorexant would not be expected to persist into subsequent waking hours at therapeutic doses less than 20 mg.

## Discussion

Timing of the pharmacodynamic efficacy of drugs targeting insomnia is critical to promote sleep maintenance throughout the resting phase and to avoid undesired next-day effects such as residual drowsiness upon waking. GABA-A receptor modulators, the current standard of care for insomnia, act by potentiating the activity of endogenous neurotransmitters and have a low threshold of receptor occupancy for *in vivo* efficacy [25]. This low threshold allows GABA-A receptor modulators to promote sleep maintenance during the resting period, but also represents a challenge to terminate their agonist activity prior to ensuing wake periods to avoid next-day effects. DORAs provide an alternative mechanism for the treatment of insomnia by inhibiting the wake-promoting activity of orexin neuropeptides. As such, a higher percentage of receptor occupancy is required to block the effect of the endogenous peptide ligand in order to promote sleep. The current studies evaluate the pharmacokinetic and receptor-binding properties that contribute to the timing of OX_2_R receptor occupancy and *in vivo* efficacy to define the potential of the DORA mechanism of action to promote sleep maintenance during the resting phase while avoiding next-day residual effects.

The capacity of orexin receptor antagonists to block orexin-induced arousal at a given time of day is dependent upon endogenous orexin neuropeptide levels. This signal window was defined by evaluating OX-A levels in two nocturnal and two diurnal species and was correlated with sleep/wake cycles in which the highest levels of wakefulness were observed during the active phase and the lowest levels during the inactive phase. As predicted, the largest observable sleep-promoting effects of DORAs in rats were greatest during the active phase when orexin levels were highest, and effects were diminished during the inactive phase, consistent with previous studies of other DORAs [10-12]. Importantly, effects of DORAs detected during the resting phase were an augmentation of normal sleep parameters, including further attenuation of active wake, promotion of SWS, and REM sleep that was either unchanged (as seen in the present studies) or improved [10,11]. Previous analysis of DORA-22-induced changes in qEEG spectral power during the inactive phase found only minimal changes in frequency distribution relative to vehicle treatment, indicating minimal disruption of qEEG patterns during the resting phase [20]. These results were supported by the observed effects of DORA-22 on resting-phase qEEG in rhesus monkeys where gamma and theta activity remained unchanged while delta frequency power was augmented from the pattern normally seen in vehicle-treated animals (see Figure 6C). In stark contrast, the effects of the GABA-A receptor modulators diazepam and eszopiclone increased high frequency gamma power and suppressed theta activity in directions that were diametrically opposed to those normally observed during the resting phase of vehicle-treated animals. These results are consistent with disrupted sleep-stage-specific qEEG spectral patterns induced by GABA-A receptor modulators, observed previously in rats [20] and humans [15]. The relatively minimal effects of DORA-22 on both sleep and qEEG spectral patterns further indicate that DORAs selectively block orexin-mediated arousal, which is at its lowest during the normal resting phase.

The etiology of insomnia is not well understood at the neurotransmitter or neuronal pathway level, and changes in orexin levels have yet to be demonstrated as a cause of insomnia. As such, translatable preclinical models for insomnia have not been developed and the studies performed in the current work only mimic the normal, or healthy condition where orexin levels are diminished during the normal inactive phase. However, orexin levels have been observed to increase with wake-related motor activity associated with forced swimming in rats [26], in patients with restless legs syndrome [27], and are diminished in narcoleptic patients based on examination of CSF levels derived from lumbar collection [28]. Persistent and significant reductions in wakefulness have been observed during the resting phase of insomnia patients in response to suvorexant treatment [17] and to SB-649868 in a situational model of insomnia [15]. These clinical observations suggest that DORAs are effective in reversing elevated orexin signaling present in insomnia patients.

**Figure 8:**
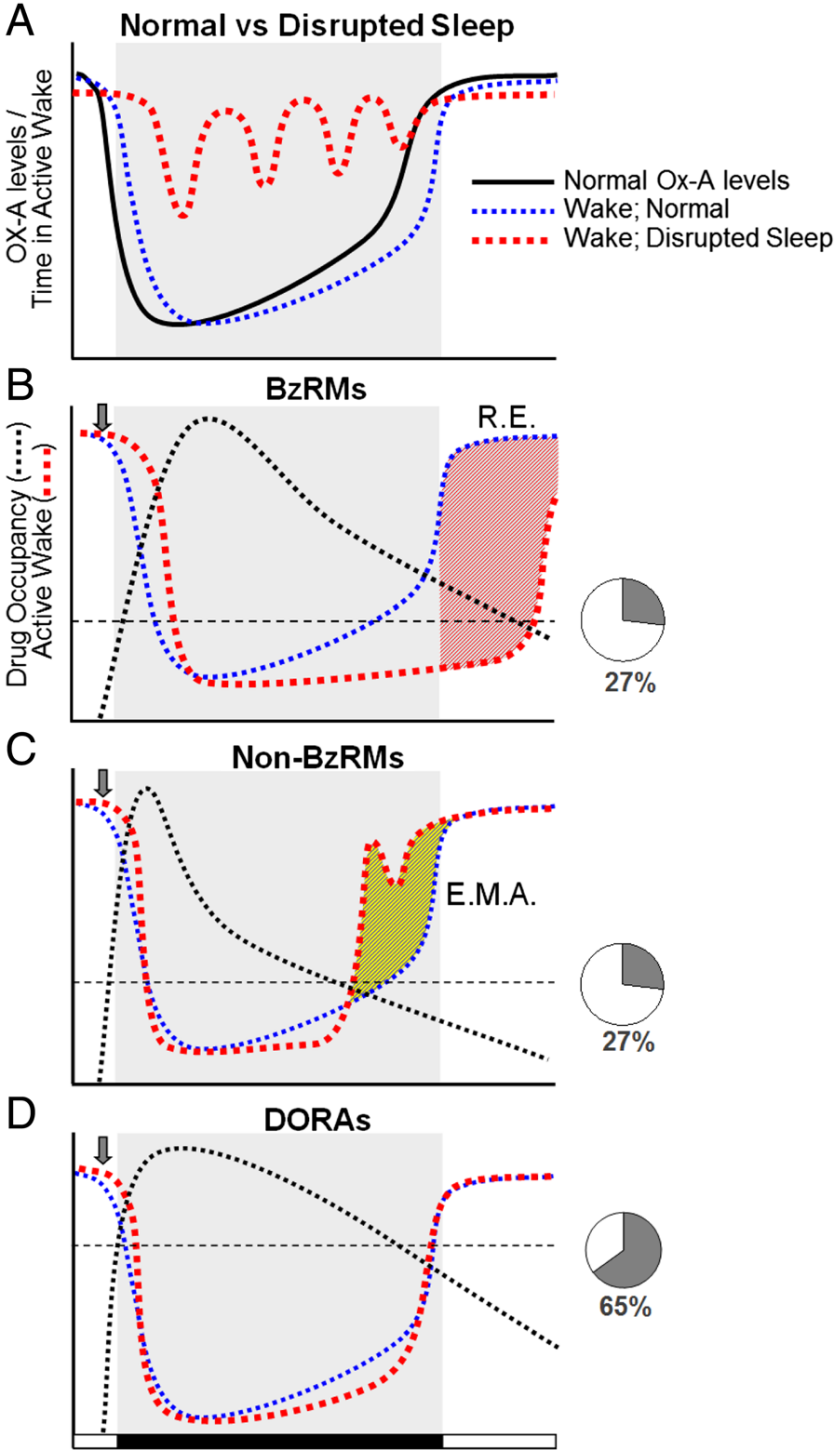
**Conceptual model for DORA OX**_**2**_**R occupancy and efficacy based on pharmacokinetic and receptor-binding properties. A**. Normal and disrupted sleep relative to OX-A levels (solid black line) during the human inactive period (gray shading). Wake during disrupted sleep (red dashed line) is punctuated by brief periods of wake after sleep onset, while normal sleep (blue dashed line) follows orexin levels. **B**. Time course of the sleep-promoting efficacy of benzodiazepine-based GABA-A receptor modulators (BzRMs). Following treatment (gray arrow), active wake diminishes as GABA-A receptor occupancy (black dotted line) exceeds that required for sleep efficacy, 27% (horizontal black dashed line) [25]. Red hatched area, difference from normal active wake (blue dashed line). Residual effects (R.E.) during the subsequent waking period as compound levels sufficient for GABA-A receptor occupancy are exceeded following the normal 8-h resting period. **C**. Efficacy time course of non-benzodiazepine GABA-A receptor modulators (non-BzRMs). GABA-A receptor occupancy (black dotted line) induced by sub-optimal doses of non-BzRMs are not sufficient to engage these receptors allowing early morning awakenings (E.M.A.). Yellow hatched area, difference from normal active wake (blue dashed line). **D**. DORAs, sufficiently engineered for optimal pharmacokinetic and receptor-binding kinetics, induce OX_2_R occupancies (black dotted line) sufficient to block the wake-promoting properties of orexin throughout the 8-h resting period and, given the high level of OX_2_R occupancy required for sleep-promoting efficacy, avoid residual effects persisting into the subsequent waking period even in the presence of moderate levels of compound exposure.

This work in rats and dogs demonstrates that salient sleep promotion by DORAs is achieved at plasma levels necessary for OX_2_R occupancy to exceed a threshold of 65 to 80% as calculated based on the transgenic rat model. Sleep promotion in dogs was observed at times during which suvorexant levels were sufficient for OX_2_R occupancies in excess of 65%. In rats, highly significant active wake attenuation in the 2 h following dosing of DORA-12 occurred at plasma concentrations corresponding to occupancies of 83% in transgenic animals, while a trend toward active wake reduction (−9.7%) was seen at an occupancy of 63% occupancies did not reach significance in this experiment. While it remains possible that variation in expression in transgenic animals expressing the human receptor may have contribute to slightly different levels of receptor occupancy, the similar *in vitro* potency of DORA-12 in humans and rats (FLIPR K_b_ = 19 nM and 25 nM, respectively; unpublished observations) indicates that the results in transgenic animals were a reasonable facsimile of receptor binding in genetically unmodified animals. The 65 to 80% occupancy range is consistent with prior studies measuring OX_2_R occupancy by orexin antagonists in specific brain regions. Although efficacy thresholds were not explicitly defined, Dugovic et al. (2009) did not observe almorexant efficacy at 78% occupancy, and appeared to see significant changes at occupancies above 89% [29], while Morairty et al. (2012) saw differential sleep drop-off when OX_2_R occupancies diminished from 75 to 63% [30]. Moreover, effective occupancy thresholds of 65 to 90% for antagonists targeting CNS G protein coupled receptors such as neuroleptics for D2-like receptors, have been found previously [31,32]. Comparisons between methods, however, should be made with caution since different approaches utilizing varying assay incubation times coupled with differing compound off-rates can influence raw occupancy measurements. Importantly, the pattern of increased OX_2_R occupancy in rats alongside dose-dependent attenuation of active wake with structurally distinct DORAs observed herein and in other published studies [10,12,18] indicates that the occupancy threshold required for efficacy is a general property of orexin antagonism and is not compound dependent. These findings illustrate the importance of mechanism-dependent differences in receptor occupancy thresholds in determining the timing of sleep-promoting efficacy of GABA-A receptor modulators relative to DORAs. Ideally, insomnia treatments are expected to normalize the elevated levels of active wake experienced during disrupted sleep that are characteristic of insomnia during the resting period (Figure 8A). GABA-A receptor modulators, including diazepam (a benzodiazepine receptor modulator, BzRM) as well as zolpidem and eszopiclone (non-BzRMs), exhibit *in vivo* efficacy at a mean GABA-A receptor occupancy of 27%. Low levels of receptor engagement are likely due to the receptor activation mechanism of these compounds leading to amplification of cellular signaling [25]. This low threshold for efficacy presents a challenge to GABA-A receptor modulators to restrict their sleep-promoting effects to the inactive phase. GABA-A receptor occupancy by diazepam, an anxiolytic BzRM with a long T_1/2_, exceeds this occupancy threshold long into the subsequent active period at sleep-promoting doses. As a consequence, diazepam exhibits substantial residual effects (Figure 8B) in terms of both sedation and cognitive measures (see Figure 6). Non-BzRMs that have a shorter exposure T_1/2_ more rapidly exceed receptor occupancy thresholds sufficient for sleep promotion. However, depending on dosage, these non-BzRMs may not maintain sleep throughout the inactive period, resulting in early morning awakenings (Figure 8C). Identifying appropriate dosages that avoid residual effects but maintain resting-phase efficacy is complicated by the additional non-sleep-related effects of these compounds. In the current studies, eszopiclone-induced reductions in active wake were detectable for up to 6 h while qEEG effects were observed almost 14 h later and effects on attention for up to 16 h (see Figure 6).

In contrast, DORAs exhibit *in vivo* sleep effects at plasma exposures sufficient for 65% OX_2_R occupancy, the higher level of receptor engagement being required to sufficiently block the activating effect of the orexin ligand (Figure 8D). This high occupancy threshold requires DORAs to maintain plasma concentrations sufficient to promote sleep maintenance throughout the normal resting period. At the same time, DORAs are less likely to exhibit next-day residual somnolence despite being present at measurable plasma levels due to the high receptor occupancy threshold required for sleep maintenance. The sleep-promoting effects of falling levels of the antagonist at the onset of the normal waking phase are also likely to be countered by accumulating levels of the endogenous orexin ligand, which might provide an even faster cessation of DORA-induced somnolence due to the competitive nature of the antagonists and the relatively close K_i_ values.

Unlike GABA-A receptor modulators, DORA-22 did not exhibit next-day residual effects in rhesus monkeys despite the presence of measurable plasma levels 17 h after dosing. DORA-22 did not impact next-day sleep, qEEG, or cognitive measures as compound levels were insufficient to occupy OX_2_R above the necessary efficacy threshold. Diazepam, an anxiolytic with a long T_1/2_, promoted sleep throughout the normal resting period and into the subsequent active phase and also impacted memory and attention. Eszopiclone did not demonstrate next-day sleep effects or memory impairment; however, it did significantly impair attention as measured by SCRT. Although sleep effects did not persist with eszopiclone, qEEG spectral power changes were evident up to lights on/performance of DMS tasks. The effect of cognitive testing on qEEG measures is currently uncharacterized, and the effect of administering a treatment for insomnia in addition to cognitive testing on qEEG spectral power is even less certain. Direct compound effects may be one possible explanation for the next-day attention deficits found with eszopiclone, as measurable concentrations (535 nM) were reported 17 h after administration. Another possibility is that this attention deficit is a consequence of abnormal sleep occurring during the previous inactive phase. Sleep-stage disruption, particularly of REM sleep, which is thought to restore cognitive performance [33,34], may be responsible for potentiating this deficit. Eszopiclone induced substantial changes in gamma and theta qEEG spectral power relative to vehicle for the entire resting period – a result consistent with both suppressed REM sleep and disrupted sleep-stage-specific EEG spectral activity observed in previous studies [15,20].

Conversely, the lack of qEEG spectral disruption by DORA-22 during the previous resting phase was associated with no such cognitive impairment. Currently, it is unclear whether the lack of cognitive impairment by DORA-22 is due to the promotion of unperturbed sleep or a lack of on-drug cognitive effects. Indeed, cognitive assessment of rhesus monkeys performed within 2 h following treatment indicated no impairment in DMS and SCRT tasks with DORA-22, but substantial deficits were induced by diazepam, eszopiclone, and zolpidem at sleep-promoting doses [24].

Even if DORA levels sufficient to exceed the OX_2_R efficacy threshold are present at the onset of wake, emerging preclinical data indicate that these effects are expected to be benign relative to those induced by GABA-A receptor modulators. When evaluated acutely (< 4 h after treatment), DORAs have little or no impact on locomotor coordination in rats [35], or on memory or attention in rats and rhesus monkeys [24]. This is in stark contrast to BzRMs or non-BzRMs, which induce substantial deficits when measured acutely [24,35]. While these differential effects remain to be investigated clinically, results in preclinical species indicate another fundamental difference between blockade of orexin signaling and GABA-A receptor activation.

The sleep promoting effect of DORAs is the result of the inhibition of orexin-induced arousal primarily mediated through OX_2_R. Genetically, loss of this receptor is associated with narcolepsy in dogs [36] and the hypersomnolent phenotype of mice lacking OX_2_R is similar to that of those lacking both receptors or *Hcrt* gene encoding orexin ligands [37,38], while OX_1_R KOs reportedly display only a minor, sleep fragmentation phenotype [39]. Pharmacologically, OX_2_R selective antagonists induce sleep promoting effects similar to that of DORAs, while OX_1_R selective antagonists administered alone exhibit only minor, if detectable effects on vigilance state [29,30]. It has been suggested that OX_1_R inhibitory activity of DORAs may counter the effect of OX_2_R inhibition by these compounds through a dopamine-mediated mechanism [29], but further evaluation using alternative OX_1_R and OX_2_R selective antagonists of different selectivity has questioned this conclusion [30].

Based on these preclinical evaluations of structurally divergent DORAs, suvorexant exhibits a pharmacokinetic profile predicted to be advantageous for the treatment of insomnia. Analysis of the time course of suvorexant plasma concentrations determined in an early Phase 1 clinical trial indicates that concentrations sufficient for efficacy in humans (0.33 μM for 65% OX_2_R occupancy based on normalization from the rat transgenic model) are restricted to normal sleep periods while efficacy is maintained for a range of doses. Given their high OX_2_R occupancy threshold for efficacy, DORAs require a plasma concentration sufficient to maintain occupancy in order to promote sleep throughout the resting period in humans. For suvorexant, this is achieved with a plasma concentration T_1/2_ exceeding 8 h. In early clinical trials, suvorexant exhibited an apparent terminal T_1/2_ of 9 to 13 h [40] – pharmacokinetic timing appropriate for sleep maintenance.

Although insomnia is a common disorder, its underlying mechanisms are still not fully understood. DORAs have been rationally designed specifically to promote sleep by blocking wakefulness and thus alleviate the symptoms of insomnia while minimizing potential off-target activity that occurs with widespread signaling via GABA-A receptor modulators. By using antagonist compounds like suvorexant to block the arousal-promoting effects of orexin signaling in the brain, the oscillation in endogenous orexin pathway activation (highest signaling during active periods and diminished signaling during normal sleep times) is modified to mimic more closely the expected physiological state in normal sleep and wakefulness.

## Conclusion

The combination of desirable pharmacokinetic and receptor-binding properties as well as a high level of OX_2_R occupancy necessary to antagonize the wake promoting effects of the endogenous orexin ligand allows DORAs to alleviate insomnia symptoms during normal sleep periods while avoiding residual somnolence during subsequent waking periods. These data support the thesis that DORAs such as suvorexant may have pharmacological and kinetic-binding properties appropriate for the treatment of insomnia.

## Methods

All animal studies were performed in accordance with The National Research Council’s Guide for the Care and Use of Laboratory Animals (http://www.nap.edu/catalog.php?record_id=12910) and were approved by the Merck Institutional Animal Care and Use Committee. All efforts were made to minimize animal use and suffering.

### Detection of orexin-A in cerebrospinal fluid

CSF was collected via cisterna magna puncture similarly from male and female beagles and rhesus monkeys (*Macaca mulatta*) similarly. Propofol (i.v., 2–8 mg/kg, to effect ) anesthetized dogs were maintained on isofluorane anesthesia via an endotracheal tube. Using aseptic technique, a 1.5 inch spinal needle with stylet is introduced through the skin and subcutaneous tissue and then through the atlanto-occipital membrane which connects the first vertebrae with the skull and overlies the cisterna magna. The stylet is then removed from the needle and cerebrospinal fluid is allowed to flow into a collection tube. CSF was collected from conscious male and female rhesus monkeys (*Macaca mulatta*) using methods described previously [41], and similar to those procedures employed in dog.

CSF was collected from Male Sprague–Dawley rats (Taconic Farms, Hudson, NY) under isofluorane anesthesia (5% with oxygen at 2 L/min in an induction hood, 2% thereafter) and on a circulating water warming blanket. Heads were shaved from the posterior portion to the dorsal thoracic area exposing region just above the cisterna magna, and placed on stereotaxic apparatus to position the head of the rat at a 70-80° bend. After identifying the area by palpation, a 25 g × 5/8” butterfly needle, with 12” of tubing (Terumo Surflo Winged Infusion Set, Terumo Corporation, Somerset, NJ) is slowly inserted into the cisterna magna of the rat. A small suction is applied from a syringe connected to the end of the winged infusion set until fluid is observed in the tubing. CSF is then collected by expulsion into a microfuge tube. CSF was similarly collected from male C57BL/6 mice (Taconic Farms, Hudson, NY) except that the procedure is a non-survival surgery in mice, and animals are rapidly euthanized following CSF recovery. For mice, a stereotaxic device is fashioned from the styrofoam 15 mL conical tube holder, and skin and muscle covering the head and neck area are removed to visualize the dura mater prior to CSF collection.

Concentrations of OX-A within CSF were quantified by immunoassay developed using the Meso Scale Discovery (MSD) electrochemiluminescence detection technology platform (Gaithersburg, MD, USA). Purified polyclonal immunoglobulin G (G-003-36, Phoenix Peptide, Burlingame, CA, USA), raised in rabbit against OX-A (amino acids 16–33) amide, was used as the capture antibody. Goat anti-human polyclonal antibody against orexin-A (N-18) (sc-26491, Santa Cruz Biotechnology Inc., Santa Cruz, CA, USA) was used as the primary detection antibody. Sulfo-Tag-labeled anti-goat antibody, raised in donkey, was used as the secondary detection antibody and was provided by MSD (R32AG-5). Assay wash buffer (R61TX-1) was also sourced from MSD. Blocking buffer was generated by dissolving membrane blocking agent (RPN-2125, GE Healthcare, Buckinghamshire, UK) in MSD wash buffer (1.5 g per 50 mL). Antibody and orexin standard diluent was made by diluting blocking buffer three-fold in wash buffer. Standard OX-A (full length peptide) was sourced from Phoenix Peptide (003–30, Burlingame, CA, USA).

Plates were incubated overnight with 25 μL of capture antibody (3.33 μg/mL) and washed three times using MSD wash buffer (200 μL per well). Plates were then incubated with blocking buffer (150 μL per well) on a Titer Plate Shaker (Barnstead International, Dubuque, IA, USA) for 1 h at room temperature. After aspirating blocking buffer, orexin standards (reconstituted in antibody diluent) or samples (25 μL) were added to each well. The plates were sealed and incubated, shaking for 2 h at room temperature. Following incubation with standards and samples, plates were washed an additional three times with 200 μL per well of wash buffer. Next, 25 μL of primary antibody dilution (1 μg per mL) was added to each well and plates were sealed and incubated while shaking for 1 h at room temperature. Following an additional wash step, 25 μL of secondary antibody dilution (0.5 μg per mL) was added to each well, and plates were sealed and incubated, shaking, for 1 h at room temperature. As a final step, 150 μL of read buffer was added to each well and plates were read on an MSD sector imager. All measurements were performed in triplicate.

### EEG

The mean time spent in sleep stages was determined in radio-telemetry-implanted mice, rats, dogs, and rhesus monkeys as first described by Renger and colleagues [42] and subsequently by Winrow and colleagues [12,18] with minor modifications. For baseline studies, recordings were taken continuously from unperturbed mice, rats, dogs, and monkeys for 6, 6, 10, or 5 days, respectively, and the mean time spent in all sleep stages in 30-min intervals were averaged and plotted onto a single 24-h period (Figure 1). Almorexant is a DORA [43] that was previously in clinical development for the treatment of insomnia [10,44]. The clinical development program for almorexant was terminated in early 2011. For rat studies in which DORA-22, DORA-12, or almorexant were administered, a balanced 3-day cross-over design was employed in which all animals were treated alternately with vehicle (20% Vitamin E d-alpha tocopheryl polyethylene glycol 1000 succinate [TPGS], orally [*per os*, p.o.]) and compound for 3 consecutive days with a 5-day intervening washout period, animals being divided randomly into either vehicle- or compound-first groups. In all cases, mean time in active wake, quiet wake, SWS, and REM sleep was determined for each 30-min interval for 3 days of each condition and averaged onto a single 24-h period. Automated sleep-stage data collection and analysis were performed as described in detail previously [12]. Statistical differences in mean time spent in active wake, quiet wake, SWS, and REM sleep between vehicle- and compound-treated groups at 30-min time points were determined by a linear mixed-effects model applied t-test.

EEG responses to diazepam (10 mg/kg, p.o. in 0.5% methylcellulose); eszopiclone (10 mg/kg, p.o. in 20% Vitamin E TPGS) and DORA 22 (30 mg/kg, p.o. in 20% Vitamin E TPGS) were evaluated in rhesus monkeys implanted with subcutaneous telemetric devices (D70-EEE; Data Sciences International, Arden Hills MN) housed in a 12:12 light cycle with lights-off at 16:30 and lights-on at 04:30. Animals were fed on a calorie-controlled diet of laboratory chow supplemented with fruit and vegetables. Water was available *ad libitum* with the exception of those monkeys trained to perform cognitive tasks, for which access was restricted for up to 4 h prior to and during cognitive testing. Treatment occurred at 14:40 (Zeitgeber time [ZT] 10:10) in a balanced 1-day cross-over design: 1 day of drug or vehicle followed by 6 days of baseline followed by 1 day of vehicle or drug. Cognition tasks were scheduled for next-day evaluation as described below. Sleep/wake architecture was scored according to vigilance state (active wake, quiet wake, delta sleep I, delta sleep II, and REM sleep) at 30-minute intervals and analyzed as previously described [24]. Spectral analysis of continuous EEG was quantified between vehicle and compound after binning continuous frequencies into canonical frequency bands (reported herein: Delta, 0.5 – 4 Hz; Theta, 4.0 – 7 Hz; Gamma, 35.0 – 100.0 Hz). qEEG values are spectral power (uV2) log transformed before analysis and averaged over 30-minute intervals, expressed as means +/− SEM, and compared with vehicle using a mixed-model ANOVA at each time point with random effects for subject and date within subject in the R statistical computing environment (cran.us.r-project.org; the R Foundation for Statistical Computing, Vienna, Austria) using a linear mixed effects model for significance testing. Significant conditional differences are indicated as tick marks at the top of the graphs (short, P ≤ 0.05; medium, P ≤ 0.01; long, P ≤ 0.001) with gray vertical bars through significantly different data points.

### OX_2_R occupancy

Occupancy of human OX_2_R in transgenic rats expressing the receptor via the rat enolase promoter was performed *ex vivo* in radioligand-binding-displacement assays essentially as previously described [21] with minor modifications. To determine the relationship between OX_2_R occupancy for a given compound over a full range of plasma and CSF concentrations, hOX_2_R-expressing rats were dosed i.v. with compound. Immediately following 30 min of i.v. administration, animals were anesthetized, sacrificed, and CSF, blood, and brain tissue were harvested and frozen for analysis. Frozen tissue was weighed and thawed coincident with homogenization. The homogenate was pelleted at 21,000 × g for 1 min and the pellet was resuspended. A binding assay was run with brain tissue and radio ligand at ~ 10 × K_d_ and filtered at 12 min (in the linear association phase). Non-specific binding was determined by the addition of 1000-fold excess unlabeled ligand. Experimental (dosed)-specific binding was compared with control (vehicle-dosed) binding ([specific total counts minus specific experimental counts]/specific total counts × 100 = percent occupancy). Raw occupancy values determined in this manner over a range of plasma and CSF values were then used to construct a normalized curve where the theoretical maximum occupancy value was defined as 100% and occupancies relative to plasma or CSF values were fit to a one-sided binding (hyperbola) curve (GraphPad Prism, San Diego, CA, USA). Plasma values determined from PSG experiments in satellite animals were then used to determine corresponding occupancy values from best fit curves for a given compound at C_max_. In separate experiments, plasma, CSF, and brain tissue was collected at time points following dosing to directly measure compound levels and corresponding OX_2_R occupancies at these times. Resulting occupancy relative to measured plasma levels of compound was fit to a normalized binding curve as described previously [21].

OX_2_R occupancy in dogs and humans was calculated based on the plasma/occupancy relation determined in rats and corrected for the free fraction of suvorexant in each species. In dogs, suvorexant plasma levels determined in satellite animals coincident with PSG studies reported by Winrow and colleagues [12] were used to calculate OX_2_R occupancy levels based on standard curves of OX_2_R occupancy in rats versus the free fraction of plasma suvorexant levels. Human free plasma levels of suvorexant sufficient for 80% OX_2_R occupancy were calculated based on extensive rat data and normalized for free fraction (rat: 1.4%; human: 1%). The plasma level of suvorexant calculated to correspond to 80% OX_2_R occupancy in humans is 0.71 μM (free + bound) [17]. Human plasma levels of suvorexant collected in Phase 1 (10, 20, 50, and 76 mg doses) and Phase 2/3 (steady state, 40 mg) clinical trials were plotted against the suvorexant plasma value calculated to correspond to the exposure required for the OX_2_R occupancy determined from standard OX_2_R occupancy versus free fraction exposure curves of preclinical species.

### Monkey Delayed Match to Sample (DMS) task

All cognition testing occurred in the animal’s home cage equipped with a touchscreen. Each DMS trial was initiated with a single ‘sample’ image (150 × 200 pixels: 1.8 inches × 2.3 inches) in one of eight colors presented at the center of a touchscreen. Evaluation began when the monkey touched the sample image, at which point the screen became blank for a retention period. Following the retention period, four choice images (150 × 200 pixels) were presented, one in each corner of the screen. One of the four choice images matched the color of the sample image, whereas the three remaining ‘distractor’ images were drawn from the pool of remaining colors. A reinforcer was provided when the monkey touched the choice image that matched the color of the sample image. Incorrect choices were not reinforced and resulted in a 5 s timeout. On completion of the trial, an inter-trial interval of 5 s was presented prior to the next trial, and failure to respond to the samples or choice images within 30 s resulted in the screen turning blank for a 5 s timeout prior to initiating another trial. Test sessions comprised 96 trials or lasted for up to 45 min. Three discrete retention intervals were titrated for each subject’s baseline performance in the absence of compound to yield performances of approximately 80–100%, 55–65%, and 35–45% of responses being correct at short, medium, and long retention intervals, and ranged from 0.25–0.5 s, 2.5–14 s, and 9–39 s, respectively. Response latencies to sample and choice images were also recorded. Values reported reflect the proportion of correct responses collapsed over all retention intervals. Differences from vehicle were examined using repeated measures ANOVA.

### Monkey Serial Choice Reaction Time (SCRT) task

Each SCRT trial was initiated with the appearance of 10 blue square ‘target’ images evenly distributed at 3-inch intervals around the perimeter of a touchscreen presented together with a centrally located circular ‘centering’ image (1.4 inch diameter circle). The task began with the monkey touching the centering image, which then turned gray. The monkey was then required to touch the centering image continuously throughout a variable pre-cue interval of 0.25-7.5 s. On completion of the pre-cue interval, one of the blue target images was cued by turning red for one of four cue durations before turning back to blue. To obtain a reward, the monkey was required to touch the target image that had been ‘cued’ red within a 5 s limited hold period. Inaccurate choices were not reinforced and resulted in a 3 s timeout. On completion of the trial, an inter-trial interval of 2 s was initiated prior to the next trial. A failure to respond to the centering image within 60 s resulted in the screen becoming blank for a 2 s timeout prior to the start of the next trial. If the monkey initiated a trial but removed its hand from the touchscreen prior to the appearance of the cue, the trial was terminated and the screen was blanked during a 2 s inter-trial interval. Sessions were terminated after 20 min. Cue location and duration were pseudo-randomly assigned. Cue duration and the target size were titrated for each subject on the basis of performance during previous baseline sessions to yield a performance of 60–80% correct responses for the trials of the shortest cue duration. The briefest cue duration varied from 0.04–0.1 s and the cues varied in size from 0.2–0.7 inches, although each cue had the same touch-sensitive area of 1.75 inches. Values reported in the current study reflect the proportion of correct responses to targets after cues of the shortest duration only.

## Authors’ contributions

ALG, CJW, JJR wrote and edited major portions of the manuscript with significant interpretive and editorial comments by all authors, particularly SDK, PJC, TMcD, and PLT Rodent, dog and rhesus orexin level determinations were performed by JB with analytical and technical contributed by LY SLG, JB, JS, AS and SVF performed rodent polysomnography studies with study design, additional data analysis and interpretation contributed by ALG, PLT, and JJR Monkey polysomnography studies were performed by JB, AS, and SJT with study design, additional data analysis and interpretation contributed by ALG, PLT, and JJR Monkey cognition study design, analysis and interpretation by SJT, JU and AS Pharmacokinetic analysis and interpretation by DC, KLY, SDK and ALG Occupancy study execution and analysis by MS, CMH with study design, additional analysis and interpretation by ALG, DC, KLY All authors’ read and approved the final manuscript.
